# Clinical characteristics of bronchopulmonary dysplasia and the risk of sepsis onset prediction via machine learning models

**DOI:** 10.3389/fped.2025.1566747

**Published:** 2025-06-27

**Authors:** Yanhua Wang, Yi Wang, Linhong Song, Jun Li, Yuanyuan Xie, Lei Yan, Siqi Hu, Zhichun Feng

**Affiliations:** ^1^ The Second School of Clinical Medicine, Southern Medical University, Guangzhou, China; ^2^ Institute of Pediatrics, Faculty of Pediatrics, The Seventh Medical Center of PLA General Hospital, Beijing, China; ^3^ National Engineering Laboratory for Birth Defect Prevention and Control of Key Technology, Beijing, China; ^4^ Beijing Key Laboratory of Pediatric Organ Failure, Beijing, China; ^5^ Department of Pediatric Cardiology, Faculty of Pediatrics, the Seventh Medical Center of PLA General Hospital, Beijing, China

**Keywords:** bronchopulmonary dysplasia, sepsis, machine learning algorithms, nomogram, prediction model

## Abstract

Bronchopulmonary dysplasia (BPD), also known as chronic lung disease, is the most common cause of respiratory morbidity in preterm infants. Sepsis plays a significant role in the pathogenesis of BPD, and the systemic inflammatory response caused by sepsis is associated with lung development, leading to simplified alveoli and abnormal vascular development, which are the histological hallmarks of BPD. In this study, we conducted a retrospective analysis of the clinical characteristics of 306 preterm infants with BPD treated at our hospital from December 2019 to December 2022. We subsequently utilized ten machine learning (ML) algorithms and used clinical features to acquire models to predict BPD with sepsis. The performance of the model was evaluated according to the mean area under the receiver operating characteristic curve (AUC), sensitivity, specificity, and accuracy. The mean area under the curve (AUC) of the best predictive model was 0.93. A nomogram for sepsis onset was developed in the primary cohort with four factors: invasive respiratory support, CRIB II score, NEC, and chorioamnionitis. By including clinical features, ML algorithms can predict BPD with sepsis, and the random forest (RF) model (sorted by the mean AUC) performs the best. Our prediction model and nomogram can help clinicians make early diagnoses and formulate better treatment plans for preterm infants with BPD.

## Introduction

Bronchopulmonary dysplasia (BPD) is usually caused by mechanical ventilation and long-term use of oxygen (1). BPD, resulting from lung injury that disrupts alveolar and pulmonary vascular development, is one of the most common causes of morbidity and mortality in preterm infants. Owing to the increased survival of extremely low-gestational-age newborns, BPD remains the most common complication associated with prematurity, and its prevalence is increasing (1–3).

BPD is a multifactorial pathology influenced by a variety of prenatal and postnatal factors that affect mothers and infants (4). BPD pathology worsens and further promotes reactive oxygen species (ROS) production, and subsequent inflammation leads to sepsis (5). If children with BPD develop an infection, this may further exacerbate the inflammatory response, thereby increasing the risk of sepsis (6).

Sepsis is a clinical syndrome involving organ dysfunction caused by a disordered host response to infection (7). Preterm infant sepsis refers to an infection involving the bloodstream in infants aged <28 days of age (8). Sepsis is divided into early-onset sepsis (EOS) and late-onset sepsis (LOS) on the basis of age at presentation after birth. EOS refers to sepsis in neonates occurring within 72 h (h) of birth (some experts use 7 days), and LOS is defined as sepsis occurring at or after 72 h of life (8–10). EOS is generally caused by an *in utero* infection or by vertical bacterial transmission from the mother during vaginal delivery, whereas LOS usually occurs not only by vertical bacterial transmission but also by horizontal bacterial transmission from healthcare providers and the environment (11). The clinical manifestations of neonatal sepsis can range from nonspecific or vague symptoms to hemodynamic collapse. The early symptoms may include irritability, lethargy, or poor feeding. Others may quickly progress to respiratory distress, fever, hypothermia, or hypotension, accompanied by poor perfusion and shock (12).

Preterm infants with BPD are more susceptible to developing sepsis because of chronic lung and airway damage. Predicting the risk of BPD with sepsis is crucial for early intervention and improving patient prognosis. Few studies have reported the establishment of predictive models in adults (13–15). However, in neonatal medicine, statistical or machine learning (ML) models for predicting patients who may develop BPD with sepsis are relatively rare. Therefore, such models need to be established to help doctors identify risks early, thereby allowing them to take preventive measures or undergo early treatment.

In this study, we conducted a retrospective analysis of the clinical characteristics of preterm infants with BPD at our hospital from December 2019 to December 2022. We subsequently used ML algorithms to identify high-risk factors for the co-occurrence of BPD and sepsis.

## Methods

### Study design and study participants

This study was approved by the Ethics Committee of the Seventh Medical Center of the PLA General Hospital (S2024-046-01) and was conducted in accordance with the Declaration of Helsinki. The studies were conducted in accordance with local legislation and institutional requirements. Written informed consent for participation was not required from the participants or their legal guardians/next of kin because of the retrospective design of the study.

The study population included extremely preterm infants with BPD who were born or admitted to the Seventh Medical Center of PLA General Hospital (Beijing, China). Trained neonatologists at our center identified the patients. The following criteria were applied to construct the initial dataset:

The inclusion criteria were as follows: very preterm infants (gestational age ≤32 weeks, >28 weeks) and extremely preterm infants (gestational age ≤28 weeks) who met the diagnostic criteria for BPD.

The exclusion criteria were as follows: very preterm infants and extremely preterm infants who died were withdrawn from treatment or discharged within 28 days after birth. Patients with genetic metabolic diseases. Infants without BPD.

The patients were divided into BPD with sepsis and BPD without sepsis groups on the basis of Doppler echocardiography results after at least 36 weeks of corrected gestation.

### Clinical feature collection

Clinical data were collected from the electronic medical records of the Seventh Medical Center of the PLA General Hospital, including maternal pregnancy factors [amniotic fluid disorders, hypertension during pregnancy, gestational diabetes mellitus (GDM), preeclampsia, placental abnormality, delivery method, placenta pathology], newborn clinical data [gestational age (GA), birth weight (BWt), sex, multifetal gestations, 1-min Apgar score (5APGAR), 10 min Apgar score (10APGAR), severity of BPD, pulmonary hypertension (PH) (early-PH and BPD-related PH), patent ductus arteriosus (PDA), severe PDA (defined as needing surgery ligation), intraventricular hemorrhage (IVH), necrotizing enterocolitis (NEC), retinopathy of prematurity (ROP), clinical risk index for babies score II (CRIB II), hospital length of stay (day), invasive respiratory support (day), etc.], related laboratory tests (platelet (PLT), C-reactive protein (CRP), white blood cell count (WBC), neutrophil count (N), hemoglobin (HGB)), signs and symptoms of infection episodes such as difficulty breathing, fever (>37.5°C), hypothermia (<36.5°C), abdominal distension, feeding intolerance, etc., and microbiological characteristics. The diagnosis of chorioamnionitis was confirmed via placental histopathology. Echocardiograms at 4–7 days of age were used to assess “early-PH” (16, 17). Other diagnostic criteria were based on related criteria (18, 19).

### Statistical methods

To filter for missing data, the missing data module in Python 3.9.12 was used. In Supplementary Figure S1, each column represents a clinical variable, and the white line represents missing data. The denser the lines in each column are, the greater the number of missing values for that variable. Detailed information regarding missing values is provided in Supplementary Table S1. We removed the antenatal corticosteroid (ANC) variable, which was missing in >25% of the observations, to facilitate and ensure study accuracy.

Continuous data are presented as the mean ± standard deviation (SD) or median (interquartile range, Q1, Q3), and intergroup comparisons of normally distributed continuous data were made via two-sample *t* tests, with the test value being the t value. Intergroup comparisons of nonnormally distributed continuous data via nonparametric tests were performed via the Mann‒Whitney *U* test, with the test value being the *Z* value. Categorical data are represented by the number of cases and percentage (%), and intergroup comparisons were made via the rank sum test, with the test value being *χ*^2^. To identify the predictors of sepsis, a variance inflation factor (VIF) was first used to test all predictors for multicollinearity, followed by the inclusion of all predictors in the model via multivariate logistic regression analysis. The odds ratios (ORs) for independent risk factors for sepsis were estimated via a stepwise selection method with a 95% confidence interval (CI). A two-tailed *P* value less than 0.05 indicated a statistically significant difference. Statistical analyses were performed via SPSS version 27.0.

Binary classification was performed using Scikit-learn (version 0.24.1) in Python (version 3.9.12). Ten ML algorithms were used to differentiate between septic BPD patients and nonseptic BPD patients. The following 10 models are used: logistic regression (LR), random forest (RF), support vector machine (SVM), decision tree (DTREE), AdaBoost (ADB), Gaussian naive Bayes (NB), linear discriminant analysis (LDA), k-nearest neighbors (KNN), gradient boosting classifier (GB), and multilayer perceptron (MLP). Considering the limited number of data samples, each classification model for the ML algorithms was built on all the data via the default parameters in the scikit-learn library. The synthetic minority oversampling technique (SMOTE) expands the number of samples in a minority class to ensure equal representation among groups in ML. The bootstrap method was used 1,000 times for internal validation. The performance of each algorithm was assessed on the basis of average sensitivity, specificity, the mean area under the receiver operating characteristic (ROC) curve, and the mean F1 of the resampled samples 1,000 times for pediatric patients with combined septic and nonseptic BPD. Receiver operating characteristic (ROC) curves were plotted via the matplotlib library (version 3.3.4) in Python as part of the internal validation process. The precise contribution (magnitude and direction) of the feature output by each classifier was determined via Shapley additive explanations (SHAPs). The SHAP values were calculated via the RF algorithm for each classifier. SHAP summary plots were visualized in Python via the Sharp library (version 0.39.0).

Nomogram charts (rms package) were drawn using the selected risk factors. The concordance statistic (C statistic) and calibration curve (rms package) were used to distinguish and calibrate the nomograms. Decision curve analysis (DCA) and a clinical impact curve (CIC) were used to evaluate the clinical utility of the model (20–22). The DCA curve and CIC (ggDCA package) were used to evaluate the effectiveness and clinical applicability of the risk prediction nomogram. Statistical analyses were performed via version 4.5.0 of the R statistical software.

## Results

### Demographic and clinical features of sepsis BPD patients and nonsepsis BPD patients

A total of 306 patients with BPD were enrolled in this study, including 177 men (57.8%) and 129 women (42.2%). The demographic and clinical features of the patients are summarized in Table 1.

**Table 1:** Clinical characteristics of infants with BPD with sepsis vs. those without sepsis and EOS vs. LOS in infants with BPD with sepsis.

Variables	Total (*N* = 306)				Total (*N* = 103)			
	Sepsis (*N* = 103)	Nonsepsis (*N* = 203)	*t*/*χ*^2^	*P*	EOS (*N* = 47)	LOS (*N* = 56)	*t*/χ^2^	*P*
Clinical variables								
GA(weeks),^a^ mean ± SD	26.82 ± 1.53	27.63 ± 1.90	−3.850	<0.001	26.85 ± 1.63	26.79 ± 1.46	−0.010	0.992
BWt(g)^a^, mean ± SD	990.65 ± 282.81	1,104.42 ± 334.13	−3.561	<0.001	997.77 ± 246.79	984.34 ± 313.54	−0.715	0.475
Sex			0.121	0.728			0.113	0.737
Male	61 (59.2%)	116 (57.1%)			27 (57.4%)	34 (60.7%)		
Female	42 (40.8%)	87 (42.9%)			20 (42.6%)	22 (39.3%)		
Csec			7.793	0.005			0.082	0.775
Yes	41 (39.8%)	114 (56.2%)			18 (38.3%)	23 (41.1%)		
No	62 (60.2%)	87 (42.8%)			29 (61.7%)	33 (58.9%)		
Singleton			0.032	0.858			0.201	0.654
Yes	70 (68.0%)	140 (69.0%)			33 (70.2%)	37 (66.1%)		
No	33 (32.0%)	63 (21.0%)			14 (29.8%)	19 (18.0%)		
Stay in the hospital (d)^a^	98.53 ± 41.75	84.67 ± 34.05	−3.591	<0.001	97.30 ± 46.09	99.57 ± 38.12	−0.868	0.386
Invasive respiratory support (d)^a^	31.64 ± 38.20	28.65 ± 19.63	−5.473	<0.001	33.78 ± 45.46	29.89 ± 41.37	−0.156	0.876
Noninvasive respiratory support (d)^a^	28.65 ± 19.63	28.26 ± 23.32	−0.649	0.516	30.13 ± 23.09	27.42 ± 17.93	−0.109	0.913
Nasal catheter oxygen inhalation (d)^a^	14.04 ± 15.64	16.79 ± 12.92	−2.957	0.003	12.55 ± 12.39	15.29 ± 17.94	−0.288	0.773
CRIB II^a^^,^^c^	9 (7,11)	6 (4,8)	−6.560	<0.001	8 (7,10)	9 (8,11)	−1.247	0.212
1APGAR^a^^,^^c^	7 (6,8)	8 (6,9)	−1.952	0.051	7 (6,8)	8 (6,8)	−0.053	0.958
5APGAR^a^^,^^c^	9 (8,9)	9 (8,10)	−2.220	0.026	9 (8,9)	9 (8,9)	−1.102	0.271
10APGAR^a^^,^^c^	9 (8,10)	9 (9,10)	−1.818	0.069	9 (8,10)	9 (8,9.5)	−0.461	0.654
PIH	14 (13.7%)	43 (21.2%)	2.484	0.115	6 (13%)	8 (14.3%)	0.033	0.856
GDM	23 (22.8%)	54 (26.6%)	0.523	0.470	10 (22.2%)	13 (23.2%)	0.014	0.906
Placental abruption	19 (18.4%)	33 (16.3%)	0.215	0.643	9 (19.1%)	10 (17.9%)	0.028	0.866
Abnormal amniotic fluid	20 (19.4%)	39 (19.3%)	0.001	0.982	8 (17.0%)	12 (21.4%)	0.317	0.573
Abnormal fetal membranes^b^	2 (1.9%)	1 (0.5%)		0.264	1 (2.1%)	1 (1.1%)		1
Umbilical cord abnormalities	16 (15.7%)	23 (11.4%)	1.121	0.290	6 (12.8%)	10 (18.2%)	0.562	0.453
Maternal age(years)	32.58 ± 5.11	32.11 ± 4.23	0.823	0.411	31.30 ± 4.73	33.68 ± 5.21	−2.288	0.024
Outcomes								
ROP			15.292	0.002			1.535	0.674
Non	31 (33.7%)	115 (58.4%）			15 (34.1%)	16 (33.3%)		
I	12 (13%)	16 (8.1%)			6 (13.6%)	6 (12.5%)		
II	30 (32.6%)	41 (20.8%)			12 (27.3%)	18 (37.5%)		
III	19 (20.7%)	25 (12.7%)			11 (25.0%)	8 (9.9%)		
Early-PH	84 (84.0%)	58 (29.1%)	6.176	0.013	38 (45.2%)	46 (54.8%)	0.012	0.913
PDA			0.859	0.651				0.028
Non	5 (4.9%)	15 (7.5%)			1 (2.1%)	5 (78.6%)		
Without surgical treatment	88 (86.3%)	169 (84.9%)			44 (95.7%)	44 (8.9%)		
Surgical treatment	9 (8.8%)	15 (7.5%)			2 (4.3%)	7 (12.5%)		
NEC	18 (17.47%)	194 (9.56%)	14.447	<0.001	5 (10.6%)	13 (23.2%)	2.802	0.094
WMI			6.676	0.036				0.681
Non	72 (69.9%)	168 (82.8%)			35 (48.6%)	37 (51.4%)		
I	23 (22.3%)	26 (12.8%)			9 (39.1%)	14 (60.9%)		
II	8 (7.8%)	9 (4.4%)			3 (37.5%)	5 (62.5%)		
BPD severity			31.646	<0.001			2.429	0.297
Mild	12 (11.9%)	66 (33.2%)			7 (14.9%)	5 (9.3%)		
Moderate	40 (39.6%)	94 (47.2%)			21 (44.7%)	19 (35.2%)		
Severe	49 (48.5%)	39 (19.6%)			19 (40.4%)	30 (55.6%)		
BPD-PH	13 (13.1%)	10 (5.0%)	6.099	0.014	4 (8.7%)	9 (17.0%)	1.482	0.223
IVH	72 (70.6%)	121 (61.4%)	2.468	0.116	33 (71.7%)	39 (70.6%)	0.053	0.817
Chorioamnionitis^b^	19 (18.4%)	3 (1.5%)		<0.001	13(27.7%)	6(10.7%)	4.877	0.027

Continuous data are presented as the mean ± SD values except for median (interquartile range, Q1, Q3); categorical data as *n*/*N* (%); the figures in parentheses are percentages. n: Number of participants. Chi-square, Fisher's exact test, and Mann–Whitney *U* test were used for discrete and continuous variables.

SD, standard deviation; EOS, early onset sepsis; LOS, late-onset sepsis; BWt, birth weight; GA, gestational age; Csec, cesarean section; 1APGAR, Apgar score at 1 min; 5APGAR, Apgar score at 5 min; 10APGAR, Apgar score at 10 min; CRIB-II, clinical risk index II for infants; PIH, pregnancy-induced hypertension; GDM, gestational diabetes mellitus; IVH, cerebral intraventricular hemorrhage; ROP, retinopathy of prematurity; PDA, patent ductus arteriosus; BPD, bronchopulmonary dysplasia; NEC, necrotizing enterocolitis; WMI, brain white matter injury; BPD-PH, BPD-associated pulmonary hypertension.

^a^ Mann–Whitney *U* test, Z value.

^b^ Fisher's exact test.

^c^ Data are shown as median (interquartile range, Q1, Q3).

**Figure 1:**
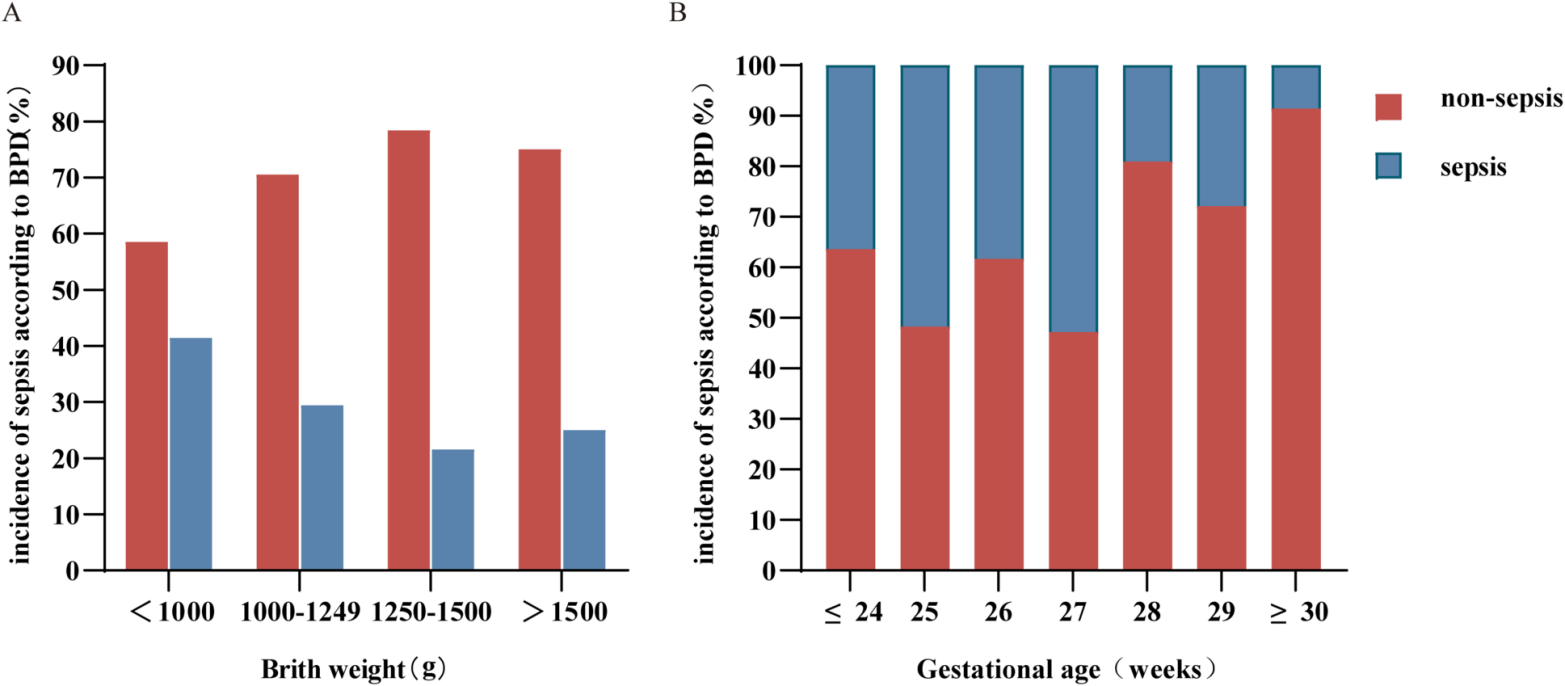
Distribution of birth weight **(A)** and gestational age **(B)** in sepsis and nonsepsis BPD patients. BPD, bronchopulmonary dysplasia.

Our analysis revealed that the lower the birth weight was, the greater the chance of developing sepsis (Figure 1A). The lower the gestational age was, the greater the incidence of sepsis, especially in preterm infants born at ≤27 weeks (Figure 1B).

The features of the patients with BPD are described in Table 1. A comparison of the two groups revealed that several characteristics, such as GA, BWt, delivery mode, hospital stay, duration of invasive ventilator ventilation, oxygen intrathecal time, CRIB II score and 5APGAR, were significantly different (*p* < 0.05). These results suggest that the earlier the gestational age and the lower the birth weight, the greater the proportion of infants with sepsis, the longer the hospital stay, and the longer the duration of invasive mechanical ventilation. Sex, singleton pregnancy, noninvasive ventilator ventilation time, and 1APGAR and 10APGAR levels were not significantly different between the two groups.

The analysis of the clinical characteristics of maternal and postnatal complications, including chorioamnionitis, varying degrees of ROP, NEC, early postnatal pulmonary hypertension (early PH), severity grading of BPD, and BPD-PH (*p* < 0.05), revealed significant differences between sepsis patients and nonsepsis BPD patients (Table 1).

### Machine learning algorithms and the development and evaluation of a nomogram for BPD patients with sepsis

In previous clinical practices, BWt, GA, invasive respiratory support, CRIB II, 5APGAR, NEC, early PH, and chorioamnionitis were found to be independent risk factors for sepsis in preterm infants with BPD. Furthermore, collinearity diagnostic analysis demonstrated that the VIFs of these risk factors, except BWt and GA, were less than 4, indicating that there was no strong indication of multicollinearity among the variables. Considering that data were missing, we included a sample size of 284 cases for model construction after removing missing data. These four variables, invasive respiratory support, CRIB II, NEC, and chorioamnionitis, were incorporated into the final predictive model on the basis of stepwise regression results. Multivariate analysis revealed that invasive respiratory support (OR, 1.02; 95% CI, 1.00–1.04; *p* < 0.05), CRIB II (OR, 1.28; 95% CI, 1.12–1.46; *p* < 0.05), NEC (OR, 3.10; 95% CI, 1.23–7.82; *p* < 0.05), and chorioamnionitis (OR, 10.40; 95% CI, 2.85–38.02; *p* < 0.05) independently increased the risk for the development of sepsis in BPD infants (Table 2).

**Table 2:** Binary logistic regression analysis of sepsis and nonsepsis patients with BPD.

Variables	OR (95% CI)	*P* value
Invasive respiratory support	1.019 (1.003–1.036)	0.022
NEC	3.098 (1.228–7.815)	0.017
Chorioamnionitis	10.400 (2.845–38.015)	<0.001
CRIB II	1.280 (1.121–1.460)	<0.001

NEC, necrotizing enterocolitis; CRIB-II, clinical risk index II for babies. *P* values were calculated via a stepwise multivariate regression model.

**Figure 2:**
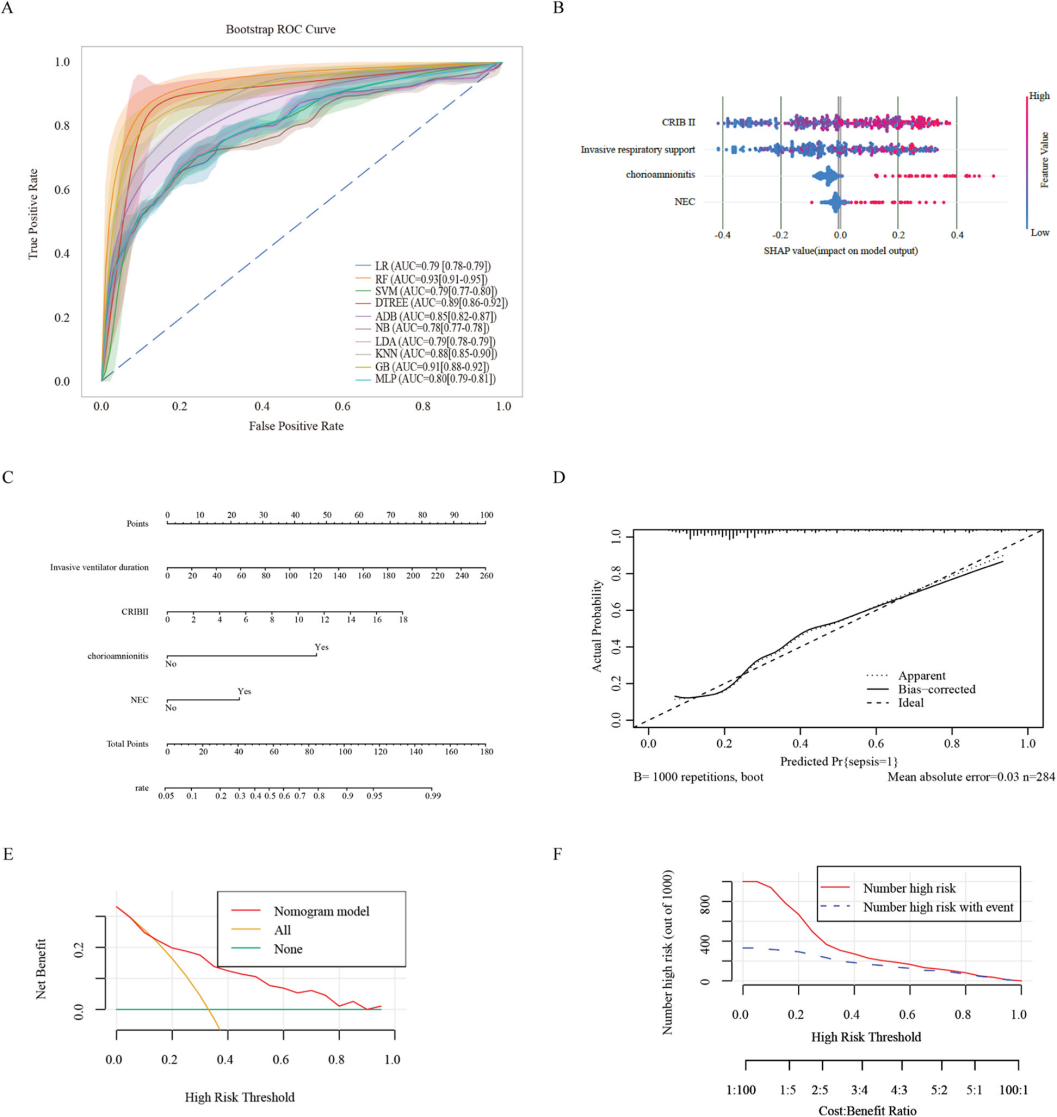
Ml and prediction models for sepsis-induced BPD patients. **(A)** ROC curves after internal validation via bootstrap resampling (1,000 times) of 10 machine learning models. the shading represents the mean AUC of the bootstrap samples, and the line represents the apparent AUC. **(B)** SHAP heatmap generated via the random forest model. **(C)** A nomogram was used to predict sepsis in infants with BPD. A binary logistic regression algorithm was used to establish the nomogram. The final score was calculated as the sum of the individual scores for each of the four variables included in the nomogram. **(D)** Calibration curve of the regression model. The *X*-axis represents the overall predicted probability of sepsis in infants with BPD, and the *Y*-axis represents the actual probability. Model calibration is indicated by the degree of fitting of the curve and the diagonal. **(E)** DCA curve of the logistic regression model. The horizontal axis in the figure represents the threshold probability, and the vertical axis represents the net benefit (NB). The lines' None “and” All “represent two extreme situations, where” None' indicates that all patients have a negative outcome, no intervention has been performed, and NB is 0. All the lines indicate that all patients have a positive outcome and that all have received intervention. Its NB is a negative sloping diagonal line. In this analysis, the decision curve provided a larger net benefit across the range of 0.2–0.80. **(F)** Clinical impact curve of the logistic regression model. LR, logistic regression; RF, random forest; SVM, support vector machine; DTREE, decision tree; ADB, AdaBoost; NB, Gaussian naive Bayes; LDA, linear discriminant analysis; KNN, k-nearest neighbors; GB, gradient boosting classifier; MLP, multilayer perceptron.

We constructed 10 models of ML by comparing their model performance. In the predictive model built with the above four factors, the mean area under the ROC curve of each model reached or approached 0.8. Among them, the RF model emerged as the most effective predictor, with a mean area under the receiver operating characteristic (ROC) curve of 0.93 (Figure 2A). The mean F1 of the 4/10 models reached 0.8, with the RF model having the best predictive performance, with a mean F1 of 0.87 (Table 3).

**Table 3:** Predictive performance comparison of the ten types of machine learning algorithms.

Models	AUC Mean, 95% CI	F1 Mean, 95% CI	Sensitivity Mean, sd	Specificity Mean, sd
LR	0.790 (0.784 0.794)	0.679 (0.650 0.706)	0.593, 0.026	0.846, 0.017
RF	0.934 (0.913 0.952)	0.877 (0.851 0.902)	0.875, 0.023	0.879, 0.024
SVM	0.794 (0.772 0.804)	0.692 (0.658 0.728)	0.622, 0.041	0.827, 0.033
DTREE	0.891 (0.858 0.919)	0.865 (0.834 0.894)	0.851, 0.025	0.885, 0.027
ADB	0.848 (0.824 0.867)	0.760 (0.718 0.798)	0.725, 0.044	0.818, 0.042
NB	0.778 (0.771 0.785)	0.619 (0.484 0.643)	0.489, 0.040	0.911, 0.016
LDA	0.791 (0.788 0.794)	0.666 (0.641 0.694)	0.568, 0.026	0.864, 0.021
KNN	0.878 (0.854 0.901)	0.810 (0.778 0.839)	0.821, 0.029	0.794, 0.031
GB	0.905 (0.885 0.925)	0.840 (0.808 0.867)	0.815, 0.030	0.874, 0.030
MLP	0.801 (0.790 0.810)	0.699 (0.663 0.730)	0.636, 0.036	0.818, 0.027

LR, logistic regression; RF, random forest; SVM, support vector machine; DTREE, decision tree; ADB, AdaBoost; NB, Gaussian naive Bayes; LDA, linear discriminant analysis; KNN, k-nearest neighbors; GB, gradient boosting classifier; MLP, multilayer perceptron. AUC, area under the curve; SD, standard deviation.

The RF model was used to demonstrate the importance of various features in the model with the best predictive performance. The CRIB II score ranked first, followed by the duration of invasive mechanical ventilation and NEC. These findings indicated that a higher CRIB II score and longer duration of invasive mechanical ventilation were associated with greater risk (Figure 2B). In addition, the coexistence of chorioamnionitis and NEC is a major risk factor for sepsis. These findings suggest that, in the absence of chorioamnionitis in pregnant mothers before birth, actively preventing prenatal infections and NEC after birth can prevent the occurrence of sepsis.

We constructed a nomogram based on the logistic regression model, which allowed for a more intuitive prediction of the risk of sepsis (Figure 2C). A nomogram for sepsis onset was developed in the primary cohort with four factors: invasive respiratory support, CRIB II score, NEC, and chorioamnionitis. These factors were screened via logistic regression analysis. The C-statistic of the nomogram was 0.79. The Brier score was 0.164, which is smaller than 0.25.

The calibration curve of the constructed regression model generated via the bootstrap method (with 1,000 repeated samples) indicated an average absolute error of 0.03, suggesting that the predicted risk of sepsis was quite accurate after calibration and that the model did not overfit (Figure 2D).

According to the DCA curve of the prediction model and the verified DCA curve (Figure 2E), the net benefit corresponding to the curve was above 0 over a wide range of decision thresholds (0.2–0.8). It is far from the two extreme curves of “None” and “All”. The CIC curves of the prediction model validation (Figure 2F) show that the results predicted by the model are close to the actual results in a wide range of risk thresholds (0.5–1.0), which indicates practical application value in a wider range of clinical situations.

### Clinical characteristics of BPD patients with EOS and LOS

Next, we analyzed the clinical information regarding birth details, postnatal complications, and symptoms from the BPD complicated with EOS group and BPD complicated with LOS group and found significant differences in the LOS group in terms of biochemical indicators (platelet count, peak values of CRP), symptoms during infection (fever, feeding intolerance, and abdominal distension), and postnatal complications (PDA) (*p* < 0.05). These findings suggest that infants with BPD and LOS are more likely to have abnormalities in the above indicators and clinical symptoms. Other factors, such as GA, BWt, the Apgar score, sex, the CRIB II score, the total SOFA score, the WBC count, HGB, postnatal complications such as NEC and early postnatal PH, and symptoms during infection, such as oxygen desaturation and bradycardia, were not significantly different between the two groups. However, in both groups of patients with sepsis, the percentage of patients with oxygen desaturation reached over 95%, whereas respiratory arrest and bradycardia were more common in patients with LOS, indicating that these patients require close attention from physicians (Table 1).

The analysis of biochemical indicators from the groups with BPD complicated by EOS and LOS revealed that the peak values of CRP were relatively high in the group with BPD complicated by LOS. In contrast, in the group with BPD complicated by EOS, the proportion of infants with abnormal white blood cell counts was relatively high (Table 4).

**Table 4:** Laboratory characteristics and clinical signs of EOS vs. LOS in infants with BPD with sepsis.

Variables	EOS (*N* = 47)	LOS (*N* = 56)	t/χ^2^	*P*
Laboratory variables				
nSOFA^a^	1 (0,2)	2 (1,4)	−1.837	0.066
WBC^b^ (10^9^/L)	16.85 ± 12.29	14.55 ± 7.74	−0.331	0.741
N^b^ (10^9^/L)	7.13 ± 6.14	10.81 ± 20.03	−0.156	0.876
HGB (g/L)	131.08 ± 26.74	122.13 ± 31.12	1.397	0.166
PLT^b^ (10^9^/L)	200.18 ± 158.55	130.43 ± 95.60	−2.412	0.016
Peak episode CRP, mg/L(median ± SD)^b^	23.71 ± 37.44	41.00 ± 37.22	−2.902	0.004
Clinical signs associated episode				
Decreased oxygen saturation	46 (97.9%)	56 (100%)	1.203	0.273
Fever or hypothermia	5 (10.6%)	15 (26.8%)	4.258	0.039
Abdominal distension/increased abdominal girth	8 (17.0%)	29 (51.8%)	13.417	<0.001
Feeding intolerance	1 (7.7%)	12 (21.4%)	8.632	0.003
Increased bradycardic episodes	8 (17%)	15 (26.8%)	1.405	0.236
Apnea	12 (25.5%)	31 (55.4%)	9.347	0.002

Continuous data are presented as the mean ± SD values except for median (interquartile range, Q1, Q3); categorical data as *n*/*N* (%); figures in parentheses are percentages.

nSOFA, sequential organ failure assessment score; WBC, white blood cell; N, neutrophil count; HGB, hemoglobin; PLT, platelet; CRP, C-reactive protein; SD, standard deviation.

^a^ The data was shown as median (interquartile range, Q1, Q3).

^b^ Mann–Whitney *U* test, Z value.

The etiological characteristic analysis of blood cultures from the groups with BPD complicated by EOS and BPD complicated by LOS revealed that in the group with BPD complicated by LOS, gram-positive bacteria accounted for 37.8%, with *Staphylococcus epidermidis* and *Enterococcus faecalis* being the main bacteria, each accounting for 16.2%, followed by Streptococcus hemolyticus and *Staphylococcus aureus*. Clinically suspected fungal infections accounted for 23.4%, and Candida spp. accounted for 3.6%. A greater proportion of infants with LOS had coinfections with more than one pathogen. In contrast, the clinically suspected rate was higher in patients with EOS (64.4%), indicating that the positive rate of pathogen detection is lower in patients with EOS and that fungal infections are rare (Table 5).

**Table 5:** Microbiological characteristics of infants and manifestations of EOS and LOS episodes [results are reported as N(%) unless otherwise specified].

Clinical diagnosis	EOS(*N* = 47)	LOS (*N* = 56)
	29 (64.4%)	7 (12.7%)
Organisms from blood culture		
Gram-nagative bacteria(total)	4 (8.7%)	16 (28.8%)
Klebsiella pnenmoniae	1 (2.1%)	8 (14.4%)
*Serratia marcescens*	-	3 (5.4%)
*Enterobacter cloacae*	2 (4.2%)	1 (1.8%)
*Enterobacter aerogenes*	-	3 (5.4%)
*Escherichia coli*	1 (2.1%)	1 (1.8%)
Gram-positive bacteria(total)	10 (21%)	25 (45.0%)
*Staphylococcus epidermidis*	5 (10.6%)	9 (16.2%)
*Staphylococcus aureus*	-	4 (7.2%)
*Enterococcus faecalis*	2 (4.2%)	1 (1.8%)
*Enterococcus faecalis* group D)	3 (6.3%)	9 (16.2%)
Hemolytic staphylococcus	-	1 (1.8%)
Staphylococcus human subspecies	-	1 (1.8%)
Fungal infection(total)	2 (4.2%)	12 (21.6%)
Pseudohyphae	-	2 (3.6%)
Candida guilliermondii		1 (1.8%)
Probable fungal infection	2 (4.2%)	9 (16.2%)
Other organisms	3 (6.3%)	3 (5.4%)
2 organisms	1 (2.1%)	3 (5.4%)
3 organisms	-	2(3.6%)

## Discussion

BPD is a common complication in premature infants and a chronic lung disease in neonates. A recent study revealed that in the United States, the incidence of chronic lung disease in extremely preterm infants born between 24 and 28 weeks of gestation has been increasing since 2012 (23). In the past decade, the survival rate of extremely preterm infants in China has improved significantly. However, the incidence of BPD has not decreased significantly, reaching 40.7% (24). In addition to chronic lung disease, the incidence of long-term complications in BPD patients, such as those involving the cardiovascular, nervous, digestive, and endocrine systems, has also increased (24).

Neonatal sepsis, especially early-onset sepsis (EOS), often presents with subtle and nonspecific symptoms, making prompt diagnosis challenging. ML models can identify high-risk neonates before symptoms become apparent. This early detection enables timely intervention, which is crucial for reducing morbidity and mortality associated with sepsis (25). ML models can integrate various clinical data points, such as vital signs and laboratory indicators, to identify risk factors more accurately. This approach significantly improves diagnostic sensitivity and overall accuracy. In addition, ML models can help allocate medical resources more efficiently by rapidly screening high-risk neonates, ensuring that critical interventions are directed toward those who need them most, enhancing the overall efficiency of healthcare delivery (26).

Inflammation/infection, including chorioamnionitis *in utero* and postnatal systemic infectious inflammation, has been shown to be a risk factor for neonatal sepsis (27, 28). Sepsis-induced systemic inflammation is a cause of neonatal mortality (29, 30).

In this study, we conducted a retrospective analysis of the clinical characteristics of preterm infants with BPD from December 2019 to December 2021 and used ML methods to rank the importance of various features and fit a predictive model. All risk factors were ranked in terms of their importance in deciding whether to include or reject them. The most important factor is the CRIB II score for neonates, followed by invasive mechanical ventilation, intrauterine infection (chorioamnionitis), and the subsequent development of acute NEC. This finding is consistent with the results of a previous questionnaire survey study (31). Early chorioamnionitis was suspected to be the main cause of EOS triggered by intrauterine infection, whereas late-onset NEC in later stages of enteral feeding was the main cause of LOS. In terms of the predictive performance of the ten models adopted, the mean area under the ROC curve of all the models can approach or reach 0.8. The RF model showed the best predictive performance, with a value of 0.93. Its clinical and practical value has been determined by DCA and CIC curves. Moreover, the nomogram constructed from these findings can intuitively predict the risk of sepsis, and when combined with the results of etiological analysis, it is beneficial to further guide clinical doctors in the diagnosis, treatment, and rational use of antimicrobial drugs. In the etiological analysis of blood for EOS and LOS in preterm infants with BPD in our hospital, gram-positive bacteria were commonly observed in LOS, and clinically suspected fungal infections, such as *Candida albicans* and *Candida parapsilosis*, were frequently observed. In this study, the changes in the platelet count and CRP level were the most significant for LOS, whereas the change in the WBC count was more pronounced for the early detection of sepsis. The platelet count has good sensitivity and specificity for diagnosing neonatal sepsis and can be used as a diagnostic tool for neonatal sepsis (32, 33). In addition, the PLT can serve as a diagnostic indicator of late-onset neonatal pneumonia (34). However, considering that CRP is nonspecific for diagnosing sepsis and has poor sensitivity, a single standard for diagnosing sepsis must be more accurate. In clinical practice, a comprehensive assessment is needed, which should be combined with clinical symptoms, blood cell counts, and other indicators.

The advent of big data and the artificial intelligence era has also driven medical progress. In recent years, the combination of ML with medical data has not only aided scientific research output but has also been continuously applied in clinical work. Statistical models are the best way to analyze and predict outcomes (35). A recent study revealed that ML models constructed from the vital signs of newborns within 24 h before infection can also predict sepsis quite well, with the area under the ROC curve reaching 0.82 (36).

In summary, our study indicated that sepsis is a risk factor for BPD. The ML algorithm suggests that the CRIB II score, duration of invasive mechanical ventilation, incidence of chorioamnionitis, and incidence of neonatal necrotizing enterocolitis are high-risk factors for the co-occurrence of sepsis. By combining the nomogram and characteristics of etiology, the risk of sepsis can be calculated, which may further reduce the exposure to and duration of antibiotic use in preterm infants and has a certain guiding significance for clinical diagnosis and treatment.

## Limitations

Our study was a retrospective case analysis study from a single center with a relatively small number of case samples. The etiological characteristics of the samples included only peripheral blood and did not include samples from other sources, such as oropharyngeal or tracheobronchial secretions or sputum. Further studies are needed to expand the sample size and to conduct prospective multicenter cohort studies. Additionally, in this study, the subjects were preterm infants, making it difficult to collect sufficient blood samples for the detection of inflammation-related indicators. In the future, given the availability of technologies that can measure these indicators, we will also incorporate these indicators into our analysis. Although ML models have shown promise in predicting sepsis, their generalizability and clinical interpretability still require further research and validation. Moreover, to increase predictive accuracy, future studies may need to incorporate more patient data and vital sign indicators as well as further refine the algorithms and structure of the models.

## Data Availability

The raw data supporting the conclusions of this article will be made available by the authors, without undue reservation.
