# Supplementary material for: Clinical characteristics of bronchopulmonary dysplasia and the risk of sepsis onset prediction via machine learning models
Source: Front Pediatr. 2025 Jun 27;13:1566747. doi: 10.3389/fped.2025.1566747 (PMC12245775; doi:10.3389/fped.2025.1566747)
Supplement: Supplementary file 1 [file Datasheet1.docx]

**Supplemental materials**


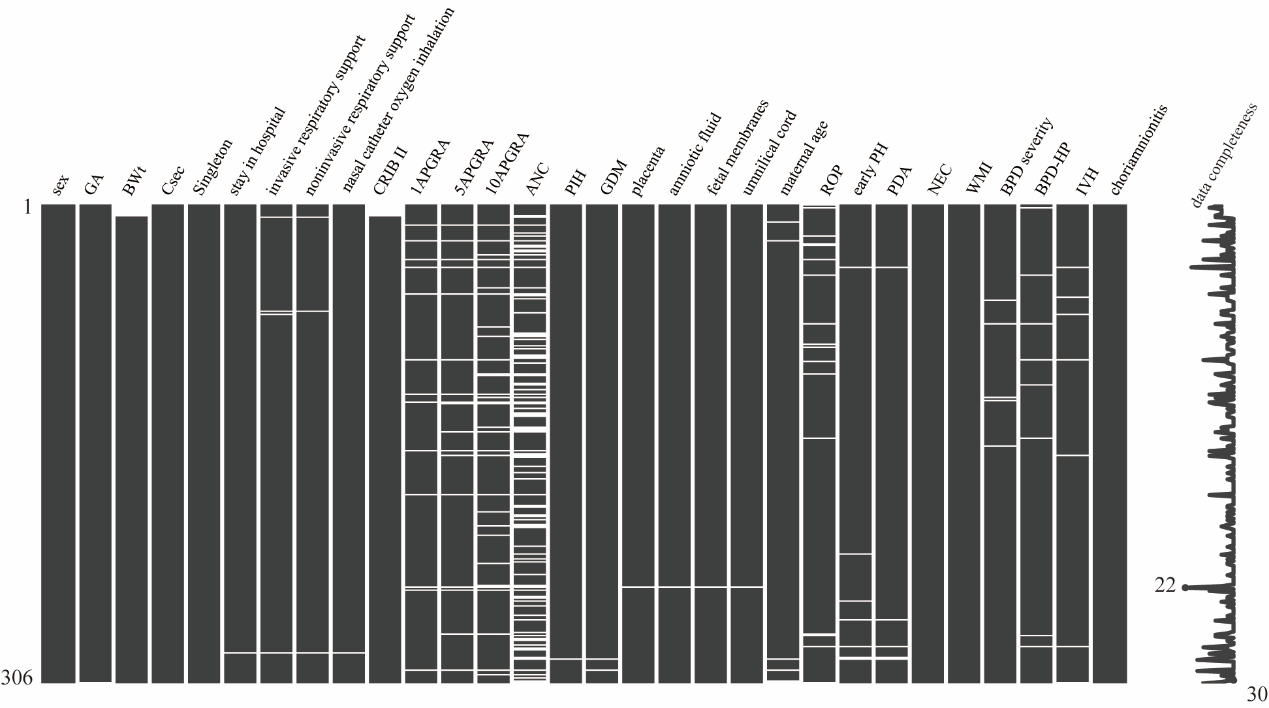


**Figure S1. Distributions of** **missing data. Each column represents a clinical variable, and the white line represents missing data.** The more white lines there are in each column, the greater the number of missing values for that variable. ANC, antenatal corticosteroid
