# Supplementary material for: Clinical characteristics of bronchopulmonary dysplasia and the risk of sepsis onset prediction via machine learning models
Source: Front Pediatr. 2025 Jun 27;13:1566747. doi: 10.3389/fped.2025.1566747 (PMC12245775; doi:10.3389/fped.2025.1566747)
Supplement: Supplementary file 2 [file Table1.docx]

**Supplemental materials**

Table S1 Missing value information

| variables | Missing values, n (%) |
| --- | --- |
| GA | 1(0.33) |
| BWt | 8(2.61) |
| Stay in hospital(d) | 1(0.33) |
| Invasive respiratory support (d) | 4(1.31) |
| Noninvasive respiratory support (d) | 3(0.98) |
| Oxygen intrathecal time in neonatal incubators(d) | 1(0.33) |
| CRIB II | 8(2.61) |
| 1APGAR | 13(4.25) |
| 5APGAR | 17(5.56) |
| 10APGAR | 31(10.13) |
| ANC | 89(29.08) |
| PIH | 1(0.33) |
| GDM | 2(0.65) |
| Placental abruption | 1(0.33) |
| abnormal amniotic fluid | 1(0.33) |
| abnormal fetal membranes | 1(0.33) |
| Umbilical cord abnormalities | 1(0.33) |
| Maternal age(years) | 6(1.96) |
| ROP | 17(5.56) |
| Early-PH | 7(2.30) |
| PDA | 5(1.63) |
| BPD-PH | 8(2.61) |
| BPD severity | 6(1.96) |
| IVH | 7(2.30) |

ANC, antenatal corticosteroid
